# Recurrent orbital inflammatory syndrome as a presenting sign of multiple myeloma: a case report

**DOI:** 10.1186/s12348-025-00463-z

**Published:** 2025-02-25

**Authors:** Chloe Depraetere, Ciel De Vriendt, Dimitri Roels, Elke O. Kreps, Virginie G. S. Ninclaus

**Affiliations:** 1 Dept of Ophthalmology, Ghent University Hospital, Ghent, Belgium; 2 Dept of Hematology, Ghent University Hospital, Ghent, Belgium

**Keywords:** Orbital inflammation, Monoclonal gammopathy, Paraproteinemia, Crystalline keratopathy

## Abstract

**Introduction:**

Orbital inflammation has a variety of underlying causes and warrants comprehensive work-up including extensive blood work and imaging studies. This case report highlights a rare presentation of multiple myeloma presenting as recurrent orbital inflammatory syndrome.

**Case Report:**

A 61-year-old woman experienced brief episodes of recurrent orbital inflammation, as documented by MRI. Work-up revealed a high-risk smoldering myeloma without sufficient evidence of plasma cell-mediated orbital inflammation on the first biopsy samples. The CRAB criteria (hyperCalcemia, Renal insufficiency, Anemia, Bone lesions) were not met, nor were there formal criteria for myeloma. The appearance of crystalline keratopathy allowed for a diagnosis of multiple myeloma, as this ocular involvement demonstrated organ damage. Myeloma treatment led to a complete and ocular remission.

**Discussion:**

This case emphasizes the importance of conducting a thorough systemic work-up and the need for repeated comprehensive ophthalmologic evaluations with each flare-up of recurrent orbital inflammation. Subtle but rare changes observed during follow-up can provide critical information that may alter the diagnosis.

## Introduction

Ocular manifestations of multiple myeloma are infrequent, but can present in a diverse range of clinical forms, affecting both the orbit and the eye. This report adds 1 more clinical presentation to the list, to raise awareness of multiple myeloma as a potential underlying cause of transient yet recurrent orbital inflammation.

## Case report

**Fig. 1:**
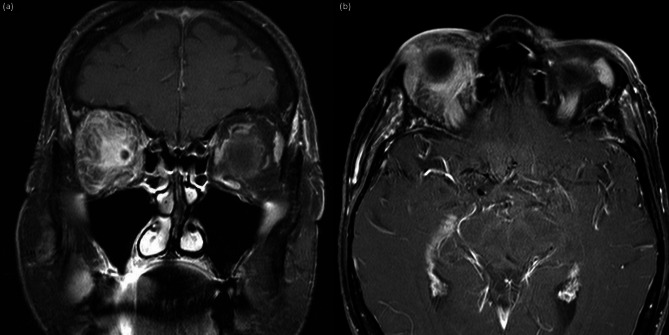
T1-weighted coronary (**a**) and transverse (**b**) MRI scan of the orbit, performed with Gadolinium contrast during the fourth episode of orbital inflammation. Notice significant enhancement and infiltration in both the preseptal and postseptal regions, including the lacrimal gland, of the right eye.

A 61-year-old woman presented with four episodes of orbital inflammation over a two-and-a-half-year period. She had no significant prior ophthalmological or oncological history. The initial episode involved the left orbit, followed by right orbital involvement seven months later. A third episode occurred in the left orbit two years after the initial presentation, and a fourth episode affected the right orbit four months after the third episode. Each episode presented with preseptal and postseptal diffuse inflammation, confirmed on CT and MRI imaging (Fig. 1). While the first two episodes resolved completely with ibuprofen 600 mg three times a day, the third episode showed symptom recurrence upon treatment discontinuation. Initial blood tests revealed normal values for blood cell counts, transaminase and kidney function, TSH, T4, TSI, CRP, erythrocyte sedimentation rate (ESR), IgG4, angiotensin-converting enzyme, and lysozyme. Indirect immunofluorescence (IFF) showed 1 + atypical fluorescence of ANCA testing non suggestive of MPO or PR3 ANCA. Further investigations revealed an IgG level of 26 g/L (reference range: 7–16 g/L) with a monoclonal kappa-type paraprotein of 21 g/L, and immunoparesis affecting IgM and IgA levels. Bone marrow biopsy demonstrated 24% plasmacytosis with atypical features, though PET CT and MRI showed no suspicious lesions. Proteinuria was absent, and the serum free light chain ratio was moderately impaired, but insufficient to meet the SLiM CRAB criteria for myeloma [1]. Orbital tissue biopsy at this time disclosed signs of mild chronic inflammation, but no evidence of lymphoma, nor plasmacytoma. A presumptive diagnosis of smoldering myeloma was made, with no indication for treatment. Additionally, the patient was diagnosed with breast cancer between the second and third episodes; however, the orbital tissue biopsy showed no signs of breast cancer metastasis.

**Fig. 2:**
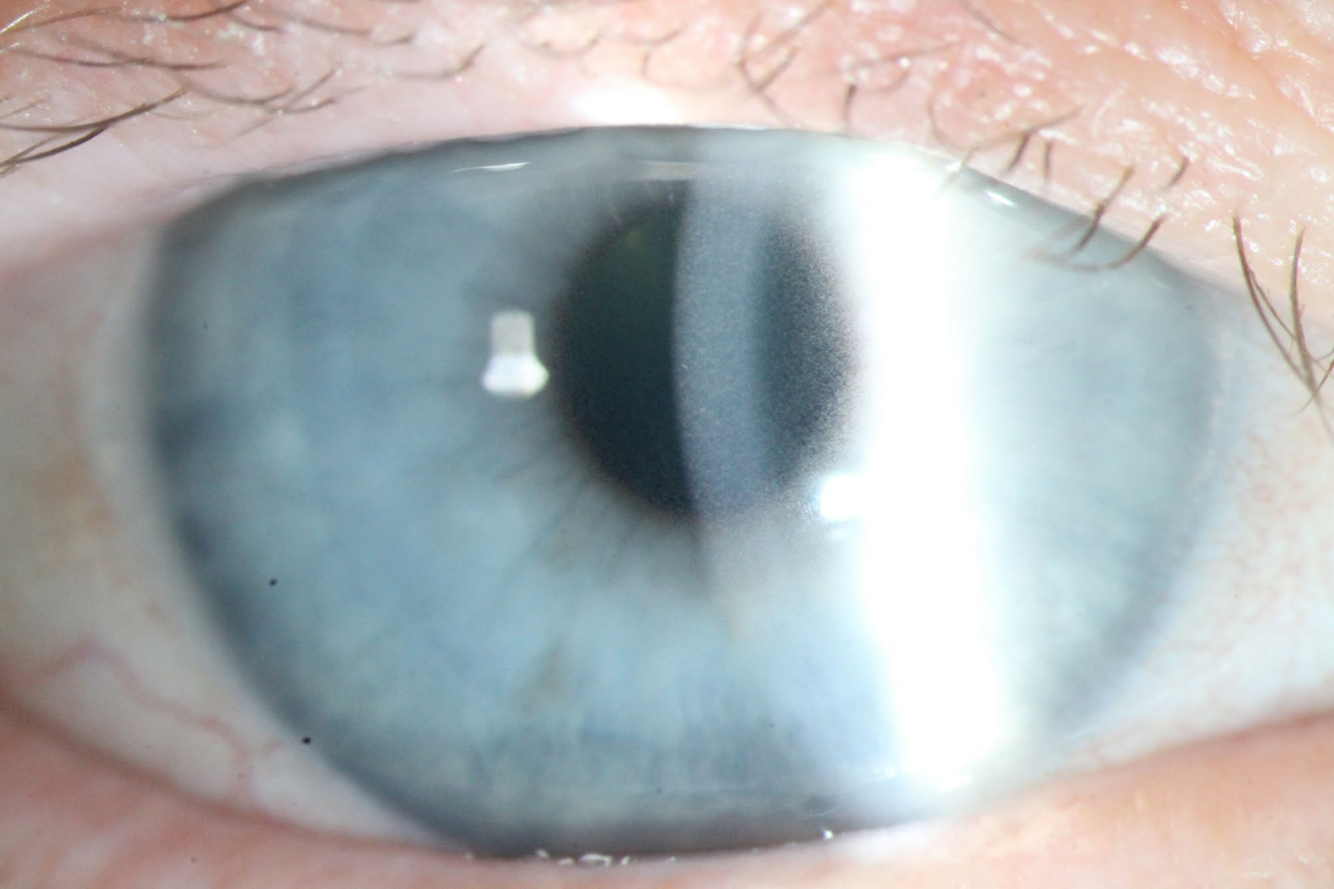
Anterior segment photography illustrating crystalline keratopathy in the right eye. Notice white crystalline deposits distributed across the entire corneal epithelium and anterior stroma.

Two and a half years after the initial presentation, during ongoing treatment for the fourth episode with ibuprofen, the clinical picture changed dramatically when the patient presented with exacerbated orbital inflammation and visual impairment. Slit-lamp examination showed a novel finding of crystalline keratopathy in both eyes (Fig. 2). This finding, highly suggestive of multiple myeloma, prompted a revision of the diagnosis from smoldering to multiple myeloma due to end-organ damage. A subsequent whole-body MRI revealed eight macroscopic osteolytic lesions, two in the ilium and six in the vertebrae, confirming the diagnosis. Systemic treatment comprising bortezomib, thalidomide, dexamethasone, and subsequent autologous stem cell transplantation in February 2022 resulted in complete remission with MRD-negativity on bone marrow biopsy. Repeat whole-body MRI after eight months showed no remaining osteolytic lesions post-treatment.

The patient’s crystalline keratopathy resolved with a combination of the systemic treatments and topical steroids, resulting in full visual recovery. After 2.5 years of ongoing follow-up since the initiation of therapy, there has been no recurrence of orbital inflammation.

## Discussion

Multiple myeloma is the uncontrolled monoclonal proliferation of plasma cells. This clonal plasma cell proliferation and its paraprotein production can give rise to a spectrum of clinical manifestations, including anemia, fractures, hypercalcemia, and renal failure [1]. The most frequent symptoms of multiple myeloma are bone pain, fatigue and recurrent infections.

Diagnostic criteria of multiple myeloma require either ≥ 10% clonal bone marrow plasma cells or a plasmacytoma on biopsy, in conjunction with monoclonal proteins detected in serum and urine electrophoresis, as well as multiple myeloma-specific clinical features [1, 2].

Orbital involvement in multiple myeloma is rare. When it does occur, it typically presents as a solitary orbital plasmacytoma. The most common clinical manifestation is proptosis, followed by decreased vision, diplopia, edema, and ptosis [3, 4]. Interestingly, a prior diagnosis of multiple myeloma before the development of orbital involvement correlates with a poorer survival rate, potentially due to the longer duration of systemic disease [4].

Beyond orbital manifestations, multiple myeloma can affect nearly every ocular structure. Documented ocular manifestations include infiltrations in the conjunctiva, uvea, and lacrimal apparatus, as well as chorioretinopathy and neuro-ophthalmologic anomalies [5, 6]. Corneal paraprotein deposition occurs in approximately 1% of patients with gammopathies. These deposits can be crystalline, amorphous, or a combination of both, and they may be present in any layer of the cornea. This characteristic distinguishes them from the crystals seen in corneal dystrophies, which are typically confined to a specific corneal layer [7]. Crystalline keratopathy may be asymptomatic or cause symptoms such as decreased vision, photophobia, irritation, or corneal epithelial defects. The precise mechanism of corneal deposition in multiple myeloma remains unclear. Some research suggests that corneal crystals may result from elevated immunoglobulin levels in the tears or aqueous humor. Alternatively, immunoglobulins might enter the cornea through limbal arteries or be supplied by keratocytes [7, 8].

To our knowledge, no other cases of multiple myeloma presenting as non-specific orbital inflammation have been reported.

Treatment of multiple myeloma has advanced significantly over the past 15 years, resulting in improved survival rates [2]. While multiple myeloma is generally not curable, therapy aims to achieve disease control and alleviate secondary symptoms. In patients without severe comorbidities, the standard treatment currently includes chemotherapy, an anti-CD38 monoclonal antibody and systemic steroids, followed by autologous stem cell transplantation. Additional treatment options encompass immunomodulatory or cellular therapy and local radiation therapy. Supportive therapy is essential for managing complications of myeloma and the adverse effects of treatment, such as pain, infections due to neutropenia and/or antibody deficiency, hypercalcemia and osteoporosis, peripheral neuropathy, and venous thromboembolism [2, 9, 10].

In conclusion, we present the first case of multiple myeloma presenting as brief episodes of orbital inflammation, occurring in rapid succession. This case study highlights the importance of a comprehensive systemic evaluation in patients with non-specific orbital inflammation, and the necessity for repeated thorough ophthalmological assessments with each recurrence of orbital inflammation. Further research is required to elucidate the underlying pathophysiology of this clinical presentation.

## Data Availability

No datasets were generated or analysed during the current study.
